# Broad-Spectrum Detection of H5 Subtype Influenza A Viruses with a New Fluorescent Immunochromatography System

**DOI:** 10.1371/journal.pone.0076753

**Published:** 2013-11-06

**Authors:** Akira Sakurai, Katsuyoshi Takayama, Namiko Nomura, Tsubasa Munakata, Naoki Yamamoto, Tsuruki Tamura, Jitsuho Yamada, Masako Hashimoto, Kazuhiko Kuwahara, Yoshihiro Sakoda, Yoshihiko Suda, Yukuharu Kobayashi, Nobuo Sakaguchi, Hiroshi Kida, Michinori Kohara, Futoshi Shibasaki

**Affiliations:** 1 Department of Molecular Medical Research, Tokyo Metropolitan Institute of Medical Science, Kamikitazawa, Setagaya-ku, Tokyo, Japan; 2 ADTEC Co., LTD. Usa-City, Oita, Japan; 3 Department of Microbiology and Cell Biology, Tokyo Metropolitan Institute of Medical Science, Setagaya-ku, Tokyo, Japan; 4 Laboratory of Microbiology, Department of Disease Control, Graduate School of Veterinary Medicine, Hokkaido University, Kita-ku, Sapporo, Japan; 5 Konica Minolta, Inc., Hino-shi, Tokyo, Japan; 6 Department of Immunology, Graduate School of Medical Sciences, Kumamoto University, Honjo, Chuo-ku, Kumamoto, Japan; Northeast Agricultural University, China

## Abstract

Immunochromatography (IC) is an antigen-detection assay that plays an important role in the rapid diagnosis of influenza virus because the protocol is short time and easy to use. Despite the usability of IC, the sensitivity is approximately 10^3^ pfu per reaction. In addition, antigen-antibody interaction-based method cannot be used for the detection of influenza viruses with major antigenic change. In this study, we established the use of fluorescent immunochromatography (FLIC) to detect a broad spectrum of H5 subtype influenza A viruses. This method has improved sensitivity 10–100 fold higher than traditional IC because of the use of fluorescent conjugated beads. Our Type-E FLIC kit detected all of the H5 subtype influenza viruses that were examined, as well as recombinant hemagglutinin (HA) proteins (rHAs) belonging to the Eurasian H5 subtype viruses and the Type-N diagnosed North American H5 subtype influenza A viruses. Thus, this kit has the improved potential to detect H5 subtype influenza viruses of different clades with both Type-E and Type-N FLIC kits. Compared with PCR-based diagnosis, FLIC has a strong advantage in usability, because the sample preparation required for FLIC is only mix-and-drop without any additional steps such as RNA extraction. Our results can provide new strategies against the spread and transmission of HPAI H5N1 viruses in birds and mammals including humans.

## Introduction

Influenza is a highly contagious respiratory disease of humans, caused by negative-strand RNA viruses of the family *Orthomyxoviridae*. Seasonal outbreaks of influenza present global health problems involving morbidity, mortality, and economic losses. Influenza A viruses are classified into 16 HA and 9 NA subtypes, based on 2 viral surface proteins–hemagglutinin (HA) and neuraminidase (NA). Almost all possible combinations of HA and NA have been isolated from aquatic birds, poultry, and other avian species. In 1997, highly pathogenic avian influenza (HPAI) H5N1 viruses were the cause of the first lethal human infection in Hong Kong [1], [2], [3]. This constituted the first identified transmission of an avian influenza virus directly from avian to human. The HPAI H5N1 virus in Hong Kong consisted of the *HA* gene that was derived from A/goose/Guangdong/1/96-like virus (H5N1; GS/GD/1) and 7 other viral gene segments derived from A/teal/Hong Kong/W312/97-like virus (H6N1; W312) [4], [5]. The GS/GD/1-linage viruses have been circulating with frequent reassortments, and one genotype, named type-Z, of 8 new H5N1 genotypes generated by reassortment became dominant in 2002 [6]. Since 2003, HPAI H5N1 viruses have spread from Asia to Europe and Africa, and have infected humans who have been exposed to infected poultry [7], [8]. Phylogenetic analysis of the *HA* gene from isolated HPAI H5N1viruses formally identified over 30 distinct sub-clades of the virus [9], [10]. A recent study showed that clades 0, 3, 4, 5, 6, 8, 9, and several second- and third-order groups from clade 2 have not been detected since 2008 or earlier [11]. The World Health Organization reported that the HPAI H5N1 virus has infected 620 individuals, causing 367 deaths (∼59% mortality) as of 15 February 2013 [12].

A recent pandemic of A/H1N1pdm in 2009 occurred following reassortment between 2 swine influenza viruses, triple-reassortant swine influenza virus and Eurasian-lineage swine influenza virus [13]. In 2011−2012, over 300 human cases of influenza A H3N2 variant virus (H3N2v) were reported [14]. H3N2v infection has been associated with exposure to swine at agricultural fairs in North America. The H3N2v contains the matrix segment of the viral gene from A/H1N1pdm [15]. These reports indicated the possibility of the occurrence of a pandemic through similar events. If this mutated HPAIV was to acquire the ability for efficient human-to-human transmission with high pathogenicity in humans, it might pose a serious threat to human health and the global economy.

Immunochromatography (IC), an antigen-detection assay completed within 20 minutes, is an important rapid test for clinical diagnosis and surveillance of influenza viruses [16], [17]. IC is quick and easy to use, but has a relatively low sensitivity. The specificity is >90%, whereas the sensitivity is approximately 60% [18]. In addition, IC is based on an antigen-based principle, liked enzyme-linked immunosorbent assay (ELISA), meaning the low sensitivity of IC could be due to low activity of the antibody to the antigen, such as HA protein which is frequently mutated. Improvement of the sensitivity and reactivity of IC would make this technique an important tool in the detection of HPAI H5N1 virus. This study establishes specific antibodies for detecting influenza virus HA of the H5 subtype and shows that IC can be improved using antibodies conjugated with fluorescent beads (Fluorescent immunochromatography; FLIC). Although the FLIC strip has to be scanned by a fluorescent reader, the sensitivity is significantly improved. In addition, our combination of antibodies can detect a broad spectrum of H5 subtype influenza A viruses. Thus, FLIC is expected to provide new strategies for initial diagnosis of the human transmission of HPAI H5N1 viruses to humans.

## Results

### Antibody characterization

**Figure 1:**
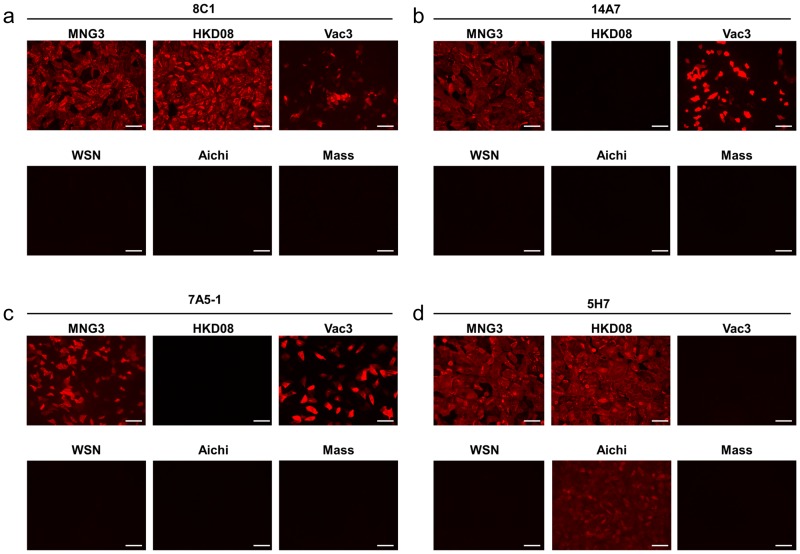
Reactivity of each antibody against H5 subtype HA. MDCK cells infected with MNG3, HKD8 or Vac3 at MOI 4. At 6-infection, cells were stained with antibody 8C1 (a), 14A7 (b), 7A5-1 (c) or 5H7 (d). Bar: 100 μm.

To determine the antigenic specificity of monoclonal antibodies, 4 antibodies of total 12 clones were selected for immunofluorescent assay (IFA) and ELISA (**Table. 1**). MDCK cells were infected with 3 H5N1 subtype avian influenza viruses: A/whooper swan/Mongolia/3/2005 (Eurasian type clade 2.2; MNG3), A/whooper swan/Hokkaido/1/2008 (Eurasian type clade 2.3.2.1; HKD08), and A/duck/Hokkaido/Vac-3/2007 (Eurasian type outlier; Vac-3) at a multiplicity of infection (MOI) of 4. To check cross reactivity, cells were also infected with influenza viruses of 3 other subtypes: A/WSN/1933 (H1N1; WSN), A/Aichi/2/1968 (H3N2; Aichi2), and B/Mass/3/1966 (Mass) at similar MOI. Infected cells were stained with candidate antibodies (**Table. 1)**. 8C1 could detect all 3 strains of H5N1 viruses (**Fig. 1a**). 14A7 and 7A5-1 could detect MNG3 and Vac-3 but not HKD08 (**Fig. 1b, c**). These 3 antibodies were not observed to interact with other subtype influenza viruses (**Fig. 1a, b, c**). 5H7 could react with MNG3 and HKD08 but not with Vac-3 (**Fig. 1d**). 5H7 showed a weak positive signal on Aichi2 infected cells (**Fig. 1d**).

**Table 1:** Specificities of Antibodies against H5 protein.

				Tested antibodies			
Method	Strain Name	Type or subtype	Genotype (clade)	8C1 (IgG_1_,κ)	5H7 (IgG_2_a,κ)	14A7 (IgG1,κ)	7A5-1 (IgG1,κ)
IFA	A/whooper swan/Mongolia/3/2005	H5N1	Eurasian (Clade2.2)	+	+	+	+
	A/whooper swan/Hokkaido/1/2008	H5N1	Eurasian (Clade2.3.2.1)	+	+	−	−
	A/duck/Hokkaido/Vac-3/2007	H5N1	Eurasian (outlier)	+	−	+	+
	A/WSN/1933	H1N1		−	−	−	−
	A/Aichi/2/1968	H3N2		−	+	−	−
	B/Mass/3/1966	B		−	−	−	−
ELISA	A/Vietnam/1194/2004	H5N1	Eurasian (Clade1)	+	+	+	+
	A/Indonesia/5/2005	H5N1	Eurasian (Clade2.1.3)	+	+	−	−
	A/bar-headed goose/Qinghai/1A/2005	H5N1	Eurasian (Clade2.2)	+	+	+	+
	A/Anhui/1/2005	H5N1	Eurasian (Clade2.3.4)	+	+	−	−

ELISA was then conducted with recombinant HA protein (rHA) of H5 subtype viruses as the target (**Table. 1**). Both 8C1 and 5H7 interacted with all 4 rHAs: A/Vietnam/1194/2004 (Eurasian type clade 1; VN1194), A/Indonesia/5/2005 (Eurasian type clade 2.1.3; IDN5), A/bar-headed goose/Qinghai/1A/2005 (Eurasian type clade 2.2; QH1A), and A/Anhui/1/2005 (Eurasian type clade 2.3.4; AH1). On the other hand, 14A7 and 7A5-1 detected rHA from VN1194 and QH1A, but not IDN5 and AH1.

### Establishment of immunochromatography using colloidal gold conjugated antibody

To determine the viability of these 4 antibodies in IC, we conjugated these antibodies to colloidal gold. First antibody combination, named Type-E, consisted of 8C1 as the immobilized antibody and 5H7 as the colloidal gold conjugated antibody. Second antibody combination, named Type-N, consisted of 7A5-1 as the immobilized antibody and 14A7 as the colloidal gold antibody. We tested 15 rHAs of H5 subtypes at a concentration of 100 or 10 ng/reaction with colloidal gold Type-E and Type-N kits (**Table. 2**). Although Type-E could detect all 13 rHAs belonging to the Eurasian type of both GS/GD/1-lineage and outlier virus, 2 North American type rHAs could not be detected. On the other hand, the reactivity of Type-N to GS/GD/1-lineage viruses was limited to clades 1, 2.1, 2.2, and these sub clades, but it could detect rHAs of Eurasian type outlier virus and North American type virus.

**Table 2:** Specificities of colloidal gold IC for H5 subtypes of recombinant HA protein.

Tested influenza A virus				Antibody combination	
Subtypes	Strain Name	genotype (clade)	Antigen amount (ng)	Type-E	Type-N
H5N1	A/Hong Kong/483/1997	Eurasian (Clade 0)	**100**	+++^a^	−
			**10**	+++	−
	A/bar-headed goose/Qinghai/14/2008	Eurasian (Clade 0)	**100**	++	−
			**10**	+	−
	A/Vietnam/1194/2004	Eurasian (Clade 1)	**100**	+++	+++
			**10**	+	+
	A/Cambodia/R0405050/2007	Eurasian (Clade 1.1)	**100**	+++	++
			**10**	++	+
	A/duck/Hunan/795/2002	Eurasian (Clade 2.1.1)	**100**	+++	+++
			**10**	+++	++
	A/Indonesia/5/2005	Eurasian (Clade 2.1.3)	**100**	+++	+
			**10**	+++	−
	A/chicken/India/NIV33487/2006	Eurasian (Clade 2.2)	**100**	+++	+++
			**10**	+++	++
	A/Egypt/2321-NAMRU3/2007	Eurasian (Clade 2.2.1)	**100**	+++	+
			**10**	++	+
	A/Turkey/1/2005	Eurasian (Clade 2.2.1)	**100**	+++	+++
			**10**	+++	+++
	A/Common magpie/Hong Kong/2256/2006	Eurasian (Clade 2.3.4)	**100**	+++	−
			**10**	+++	−
	A/Anhui/1/2005	Eurasian (Clade 2.3.4)	**100**	+++	−
			**10**	+++	−
	A/goose/Guiyang/337/2006	Eurasian (Clade 4)	**100**	+++	−
			**10**	+++	−
H5N2	A/American green-winged teal/California/HKWF609/2007	North American	**100**	−	+++
			**10**	−	+
H5N3	A/duck/Hokkaido/167/2007	Eurasian (outlier)	**100**	+++	+
			**10**	+	+
H5N8	A/duck/NY/191255–59/2002	North American	**100**	−	+++
			**10**	−	+

a The number of + indicates the level of signal. +, ++ or +++ indicates the intensity of signal is between 105 and 125%, between 126 to 175% or over 176% of that of background, respectively.

To evaluate the ability of IC to detect virus, 3 H5N1 viruses, MNG3, HKD08 and Vac-3, were tested by using Type-E and Type-N kits at serial concentrations (**Table. 3**). Type-E detected all 3 viruses with a sensitivity between 10^5^ and 10^6^ pfu (per 100 μl sample solution), and Type-N detected MNG3 and Vac-3 with a sensitivity of about 10^5^ pfu (per 100 μl sample solution), but not HKD08. Interestingly, 5H7 could not detect Vac-3 under the condition of IFA, but Type-E, which consisted of 5H7 as the colloidal gold conjugated antibody, could detect Vac-3, indicating that 5H7 is more suitable for detection of HA proteins of Eurasian type outlier under the conditions of IC than IFA.

**Table 3:** Specificities and sensitivities of colloidal gold IC for H5 subtypes of influenza A viruses.

Tested H5N1 influenza virus			IC type	
Genotype (clade)	Strain Name	Viral titer (pfu/reaction)	Type-E	Type-N
Eurasian (calde2.2)	A/whooper swan/Mongolia/3/2005	10^6^	++^a^	+++
		10^5^	+	++
		10^4^	−	−
Eurasian (Clade 2.3.2.1)	A/whooper swan/Hokkaido/1/2008	10^6^	++	−
		10^5^	−	−
		10^4^	−	−
Eurasian (outlier)	A/duck/Hokkaido/Vac-3/2007	10^6^	+	+++
		10^5^	−	+
		10^4^	−	−

a The number of + indicates the level of signal. +, ++ or +++ indicates the intensity of signal is between 105 and 125%, between 126 to 175% or over 176% of that of background, respectively.

### Establishment of FLIC for the detection of H5 subtype viruses

**Figure 2:**
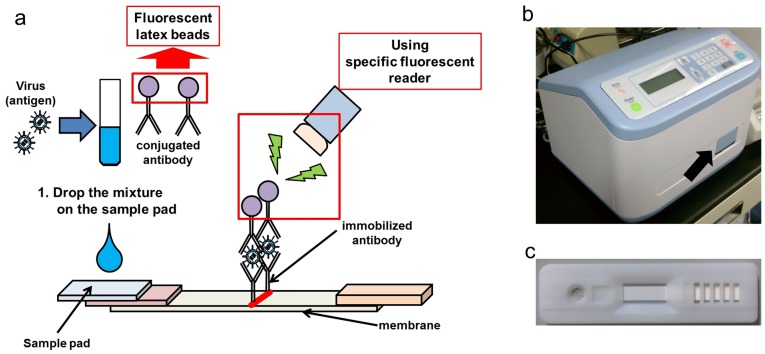
Principles and appearance of FLIC. (a) Fluorescent latex beads were conjugated to the detection antibody and the signal was detected by the specific fluorescent reader. (b) Photograph of the fluorescent reader. The arrow indicates the insertion slot for FLIC strip. (c) Photograph of the FLIC strip.

**Figure 3:**
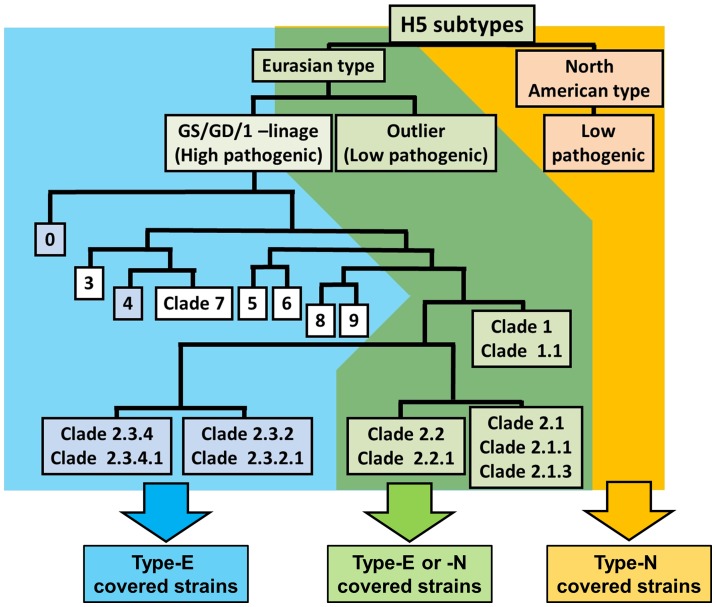
Schematic diagram of phylogenetic tree of H5 subtype HA protein and detectable area by each type of FLIC. White boxes indicate that no virus belonging to the clade was determined, and the boxes with a number only indicate that the clades have not been detected since 2008 or earlier.

Because in the present experiment, it was indicated that the 2 kits have the potential to detect HA protein of the H5 subtypes, establishment of FLIC for detection of H5 subtype viruses was undertaken to improve IC. The principles of FLIC were shown in **Fig. 2**. 5H7 and 14A7 were conjugated with fluorescent beads for FLIC, instead of colloidal gold. Type-E and Type-N of FLIC were tested for detection of 10^6^ 50% tissue culture infectious dose (TCID_50_) of 7 strains of H5N1 viruses and 2 strains of H5N2 viruses (**Table. 4**). Type-E could detect all 7 Eurasian type viruses both GS/GD/1-lineage and outlier, but not 2 North American type viruses. On the other hand, FLIC Type-N detected only 5 strains: MNG3, Vac-3, A/Muscovy duck/Vietnam/OIE-559/2011 (Eurasian type clade 1.1; OIE559), and 2 strains belonging to the North American type. Taken together, our results indicated that the set of Type-E could detect all Eurasian type viruses we tested but not any North American type viruses, and that the pair of Type-N detected a part of GS/GD/1-lineage (only clade 1, 2.1, 2.2, and these sub clades), Eurasian type outlier, and all North American type viruses we tested (**Fig. 3**). The combination of Type-E and Type-N is expected to have the potential to detect almost all H5 subtype influenza viruses.

**Table 4:** Specificities of H5 FLIC for H5 subtypes of influenza A viruses.

Tested influenza A virus			FLIC type		
Subtypes	Strain Name	Genotype (clade)	Type-E	Type-N	Reference
H5N1	A/Hong Kong/483/1997	Eurasian (clade 0)	+	−	[32]
	A/Muscovy duck/Vietnam/OIE-559/2011	Eurasian (clade 1.1)	+	+	AB636524
	A/whooper swan/Mongolia/3/2005	Eurasian (clade 2.2)	+	+	[33]
	A/whooper swan/Hokkaido/1/2008	Eurasian (Clade 2.3.2.1)	+	−	[34]
	A/whooper swan/Hokkaido/4/2011	Eurasian (Clade 2.3.2.1)	+	−	[35]
	A/peregrine falcon/Hong Kong/810/2009	Eurasian (Clade 2.3.4)	+	−	[36]
	A/duck/Hokkaido/Vac-3/2007	Eurasian (outlier)	+	+	[28]
H5N2	A/chicken/Taiwan/1209/2003	North American	−	+	[37]
	A/duck/Pennsylvania/10218/1984	North American	−	+	[38]

The sensitivity of FLICs was calculated using the exponential approximation of viral titer (pfu/mL) and the rate of signal/background (S/B). The cut-off S/B value was set at 1. The amount of viral titer at the intersection point of the exponential approximation and S/B  = 1 was defined as the limit of detection (LOD). The LODs of FLICs for detection of H5N1 viruses are described in **Table. 5**. Compared with colloidal gold IC (**Table. 3**), the sensitivity of FLIC is several 10 times higher than that of IC.

**Table 5:** LODs of H5 FLIC for H5 subtypes of influenza A viruses.

Tested H5N1 influenza virus		FLIC type (pfu)	
genotype (clade)	Strain Name	Type-E	Type-N
Eurasian (calde2.2)	A/whooper swan/Mongolia/3/2005	8.54×10^3^	6.28×10^3^
Eurasian (Clade 2.3.2.1)	A/whooper swan/Hokkaido/1/2008	5.77×10^4^	No signal
Eurasian (outlier)	A/duck/Hokkaido/Vac-3/2007	7.43×10^4^	2.63×10^3^

Improvement in sensitivity may cause a decrease in the specificity of the system. To confirm the specificity of FLICs, 10^7^ TCID_50_ of other subtype viruses, including HPAI H7N7 virus (A/chicken/Netherlands/2586/2003), were tested (**Table. 6**). In addition, 1 μg/mL of 15 rHA of other subtypes, including Spanish influenza virus (A/BrevigMission/1/1918) and H1N1pdm (A/California/7/2009), were also tested (**Table. 7**). All of the viruses and rHAs were not detected with any positive signals, meaning the FLICs maintained the specificity with the high sensitivity.

**Table 6:** Specificities of H5 FLIC for non-H5 subtypes of influenza A viruses.

Tested influenza A virus		FLIC type	
Subtypes	Strain Name	Type-E	Type-N
H1N1	A/WSN/1933	-	-
	A/PR/8/1934	-	-
	A/duck/Tottori/723/1980	-	-
H2N2	A/Adachi/2/1957	-	-
H3N2	A/Aichi/2/1968	-	-
H3N8	A/duck/Mongolia/4/2003	-	-
H4N6	A/duck/Czech/1956	-	-
H6N2	A/Turkey/Massachusetts/3740/1965	-	-
H7N7 HPAIV	A/chicken/Netherlands/2586/2003	-	-
H7N7	A/seal/Massachusetts/1/1980	-	-
H8N4	A/turkey/Ontario/1968	-	-
H9N2	A/turkey/Wisconsin/1966	-	-
H10N7	A/chicken/Germany/N/1949	-	-
H11N6	A/duck/England/1/1956	-	-
H12N5	A/duck/Alberta/60/1976	-	-
H13N6	A/duck/Siberia/272PF/1998	-	-
H14N5	A/mallard/Astrakhan/263/1982	-	-
H15N8	A/duck/Australia/341/1983	-	-
H16N3	A/black-headed gull/Sweden/5/1999	-	-

**Table 7:** Specificities of H5 FLIC for non-H5 subtypes of recombinant HA protein.

Tested recombinant HA protein of influenza virus		FLIC type	
Subtypes	Strain Name	Type-E	Type-N
H1N1pdm	A/California/7/2009	-	-
H1N1	A/BrevigMission/1/1918	-	-
H2N2	A/Japan/305/1957	-	-
H3N2	A/Brisbane/10/2007	-	-
	A/Wisconsin/67/X-161/2005	-	-
H4N6	A/mallard/Ohio/657/2002	-	-
H6N1	A/northern shoveler/California/ HKWF115/2007	-	-
H8N4	A/pintail duck/Alberta/114/1979	-	-
H9N2	A/Hong Kong/1073/1999	-	-
H10N3	A/duck/Hong Kong/786/1979	-	-
H11N2	A/duck/Yangzhou/906/2002	-	-
H12N5	A/green-winged teal/ANB/199/1991	-	-
H13N8	A/black-headed gull/Netherlands/ 1/2000	-	-
H15N8	A/duck/Australia/341/1983	-	-
H16N3	A/black-headed gull/Sweden/5/1999	-	-

## Discussion

Clinical diagnostic tests for influenza viruses in outpatient departments or clinics are typically based on IC detection of influenza virus antigens [19]. Basically, diagnosis of influenza is made by detection of NP protein as a target antigen. On the other hand, for identification of the H5 subtype, it is necessary to detect the HA protein, which is in much lower abundance than the NP protein. This indicates that the sensitivity of IC would have to be improved. Additionally, IC is based on an antigen–antibody reaction, implying that it is not suitable for detecting emerging or re-emerging influenza viruses that are precursors of epidemics or pandemics. Thus, our goals for this study were to improve sensitivity of IC and establish a pair of antibodies that efficiently covers almost all H5 subtype HAs.

In this study, we succeed in improving IC by using antibodies conjugated with fluorescent beads and a specific IC reader. The sensitivity of FLIC was several 10 times higher than that of colloidal gold IC (**Tables 3** **and** **6**), indicating that FLIC is one of the possible approaches for improving the diagnosis of disease caused by any pathogens. FLIC has additional advantages over IC, including convenience and rapidity, but one disadvantage is the requirement of the specific machine. Nevertheless, the FLIC reader provides objective measurement of positive signals. The LODs of Type-E and Type-N FLIC are 8.54×10^3^ pfu or 2.63×10^3^ pfu (per 100 μl sample solution), respectively, and these are almost equal to the LODs of commercial IC kits detecting viral NP protein (10^2^-10^4^ TCID_50_/reaction) [17]. These results reflect that HA protein is less abundant than NP protein.

In 2013, World Health Organization has defined new 4 phases (Interpandemic phase, Alert phase, Pandemic phase and Transition phase) of the pandemic stage of a disease [20]. Alert phase is defined as the time when influenza caused by a new subtype has been identified in humans. At Alert phase, rapid identification of the new strain in humans and poutry is essential for regulatory preparedness. FLIC can play a critical role in diagnosing infected patients and poutry at Alert stage, because this method is more sensitive than the standard ICs as well as rapid and easy-to-use. FLIC can also be applied to detect other rapidly spreading pathogens, such as Middle East respiratory syndrome coronavirus [21], [22] for regulating the spread of such viruses.

Our combination of antibodies against H5 HA detected many kinds of HA proteins. Type-E could detect all influenza viruses and rHAs belonging to the Eurasian type of both GS/GD/1-lineage and outlier. Because avian influenza viruses of Eurasian type play an important role in the human infection by avian influenza viruses, the Type-E would be useful for clinical diagnosis and surveillance in Asian countries. The antisera against clade 2.3.2 viruses very weakly interact with clade 2.3.4 viruses, but that against clade 2.3.4 viruses could not detect clade 2.3.2 virus [23]. However, our Type-E kit could detect both clade 2.3.2 and clade 2.3.4 viruses, meaning the kit identifies the general epitope of Eurasian type H5 viruses. Recently, 4 HPAI H5N2 viruses were reported in China [24]. Two of these HPAI H5N2 viruses contained the HA gene belonging to clade 2.3.4. The other 2 isolates included the HA gene of clade 7. Thus, we are interested in the reactivity of Type-E to clade 7 HA. Because Type-E detected a clade 4 rHA (A/goose/Guiyang/337/2006), which is genetically close to clade 7 HA, there is a high possibility of clade 7 HA recognition by Type-E.

Type-N could detect all low pathogenic avian influenza H5 virus (LPAI H5 virus) and rHAs which we tested. All LPAI H5 viruses in poultry are notifiable to Office International des Epizooties (World Organisation for Animal Health; OIE) [25], due to the risk that LPAI H5 virus gains a high pathogenicity by mutation in poultry hosts. Ito *et al*. reported [26] that LPAI H5 virus (A/whistling swan/Shimane/499/83 (H5N3)) became highly pathogenic in chickens after 24 consecutive passages by air sac inoculation. The result indicated that LPAI H5 viruses have a potential of becoming HPAI viruses and public health threat. Thus, not only HPAI H5N1 viruses but also LPAI H5 viruses as well should be surveyed in poultry. In the above basis, our FLIC Type-N kit can become a powerful tool for rapid surveillance of LPAI H5 viruses.

We have previously reported another method for rapid diagnosis, super high-speed quantitative PCR (SHRT-PCR) [27], which is a recently developed version of qRT-PCR that is characterized by extremely short reaction times (less than 20 min per run for 40 cycles). Compared with SHRT-PCR, FLIC has a strong advantage in usability, because the work required for FLIC is only mix-and-drop without any additional steps such as RNA extraction. Thus, FLIC is more suitable not only for diagnosis in small clinics and hospitals, but also for rapid detection of HPAIV from infected-candidates in Asian countries.

## Materials and Methods

### Ethics Statement

All animal care and experimental procedures were performed according to the guidelines established by the Tokyo Metropolitan Institute of Medical Science Subcommittee on Laboratory Animal Care; these guidelines conform to the Fundamental Guidelines for Proper Conduct of Animal Experiment and Related Activities in Academic Research Institutions under the jurisdiction of the Ministry of Education, Culture, Sports, Science and Technology, Japan, 2006. All protocols were approved by the Committee on the Ethics of Animal Experiments of the Tokyo Metropolitan Institute of Medical Science (Permit Number: 11–065). All efforts were made to minimize the suffering of the animals.

### Cells and virus strains

Madin–Darby canine kidney cells (MDCK cells; American Type Culture Collection, ATCC, VA, USA) were maintained in Dulbecco's modified Eagle's medium (D-MEM) supplemented with 10% fetal calf serum (FCS) and penicillin-streptomycin solution. RK-13 cells (ATCC) were maintained in modified Eagle's medium (MEM) supplemented with 5% FCS and penicillin-streptomycin solution. Cells were grown in an incubator at 37°C under 5% CO_2_.

All influenza A virus strains used in this study are listed in Tables 3 (H5 subtypes) and 6 (non-H5 subtypes). A/WSN/33 (H1N1), A/PR8/34 (H1N1), A/Aichi/2/68 (H3N2), B/Mass/3/66, and B/Tokyo/15480/08 were described previously [27]. A/Duck/Hokkaido/Vac-3/07 (H5N1), a low pathogenic H5N1 subtype vaccine strain, was generated by genetic reassortment between 2 low-pathogenic avian influenza viruses [28]. All viruses were grown in MDCK or 10-day-old embryonated chicken eggs.

### Recombinant HA protein and antibody

All rHAs except QH1A rHA were obtained from Sino Biological Inc. (Beijing, China).

QH1A rHA was generated by recombinant vaccinia virus (rVV) [29]. A synthesized QH1A gene with 6× His tag sequence (Sloning BioTechnology, Puchheim, Germany) was inserted into pBMSF7C vector, thereby generating pBMSF7C-mCl2.2. RK13 cells derived from rabbit kidney were infected at MOI 10 with rVV LC16mO strain, followed by transfection with pBMSF7C-mCl2.2. After 36 h, the virus-cell mixture was harvested by scraping, and frozen at −80°C until use. The recombinant virus was purified and named RVV-Flu HA (H5-mCl2.2). Then, RK13 cells were inoculated at MOI 10 with RVV-Flu HA (H5-mCl2.2). Twelve hours post-inoculation, cells were washed with PBS and lysed in HA lysis buffer (20 mM Tris-HCl, pH 7.9, 500 mM NaCl, 20 mM imidazole, 10% glycerol, and 1% NP-40) containing 1 mM PMSF and 2 g/mL of aprotinin, leupeptin, and pepstatin A. The cell lysates were sonicated, and soluble extracts were prepared by centrifugation at 9,100×*g* for 30 min. His-tagged (6×) QH1A rHA protein was then purified by using Ni-NTA Agarose (Qiagen, Hilden, Germany) according to the manufacturer's instruction.

8C1, 5H7, and 14A7 were established by GANP mice with Balb/c background [30], and 7A5–1 was established by a normal Balb/c mouse. QH1A rHA was immunized to corresponding mouse with Complete Freund's Adjuvant for initial injection and Incomplete Freund's Adjuvant for boosts. Three days after the final boost, spleen cells were obtained for cell fusion by polyethylene glycol method with mouse myeloma cell line P3U1 under the standard procedure [31]. The fused cells were selected with hypoxanthine/aminopterin/thymidine medium on 96-well plates at a concentration of 2×10^4^ cells/well with IL-6 (0.25 U/mL). Hybridomas were cloned by limited dilution and several clones were picked up. The antibodies were purified from the culture supernatant by protein-A sepharose (GE Healthcare UK Ltd, England) in the accompanying manuscript.

### IFA and ELISA

For IFA, MDCK cells were inoculated with at MOI 4 with corresponding viruses for 30 min and washed with PBS several times. Fresh medium with 10% of FCS (without trypsin) was added to each well and cells were incubated at 37°C. At 6 h post infection, cells were fixed in 4% paraformaldehyde. Fixed cells were treated with each antibody for 1 h. Cy3-conjugated anti-mouse antibody (Jackson ImmunoResearch Laboratories, Inc., PA, USA) was used as a secondary antibody. Cells were observed by a fluorescence microscope (BZ-9000, Keyence, Osaka, Japan).

For ELISA, 100 ng of target rHA protein was immobilized to F96 MAXISORP NUNC-IMMUNO PLATE (Thermo Scientific, FL, USA) and plates were incubated overnight at 4°C. The immobilized plate was blocked with 150 mL of 1% BSA/PBS for 2 h at room temperature. Diluted antibody was added and incubated for 1 h at room temperature. HRP-conjugated anti-mouse IgG specific antibody was used as secondary antibody. OPD was added as a substrate for HRP, and absorbance was measured at 492 nm by ARVO™ X2 (Perkin Elmer, MA, USA).

### Development of FLIC for H5 detection

Detection antibodies (5H7 and 14A7) were conjugated with fluorescent latex beads (3602–613, Fujikura Kasei Co., LTD, Tokyo, Japan). 1 ml of latex beads solution (0.5% in 10 mM HEPES-NaOH (pH 7.0) was combined into 300 μl of water soluble carbodiimide (1 mg/ml in 10 mM HEPES-NaOH (pH 7.0), 346–03632, DOJINDO Molecular technologies, Inc., Kumamoto, Japan), and incubated for 15 min at room temperature. Antibody (0.3 mg) was added, and incubated for 1 h at room temperature. Centrifuged and discard the supernatant. Latex beads were blocked with 1% BSA solution for 1 h at room temperature. Centrifuged and discard the supernatant. Latex beads were washed with wash buffer (0.1% Tween-20, 0.1% BSA, 10 mM HEPES-NaOH (pH 7.7)), resuspended into reaction buffer (0.1% BSA, 10 mM HEPES-NaOH (pH 7.7)), and incubated for 48 h at 37°C. The fluorescent reagent was 2-[3-Chloro-5-[1,1-dimehtyl-3-(3-methyl-butyl)-1,3-dihydro-benzo [e]indol- 2-ylidene]-penta-1,3-dienyl]-1,1-dimethyl-3-(3-methyl-butyl)-1H-benzo [e] indolium hexafluorophosphate. The absorption maximum and fluorescent maximum were 683 nm and 701 nm, respectively.

Sample solutions (10 μl) were suspended in 90 μL of FLIC dilution buffer (50 mM Tris-HCl (pH = 7.2), 150 mM NaCl, 1% Trition X-100), and dropped on the sample pad of the IC strip (Fig. 1a). The fluorescence was detected with Konica Minolta immunochromatography reader (Konica Minolta, Inc., Tokyo, Japan) at excitation wavelength 660 nm and emission wavelength 710 nm (Fig. 2b).
